# Supplementary material for: MAPCap allows high-resolution detection and differential expression analysis of transcription start sites
Source: Nat Commun. 2019 Jul 30;10:3219. doi: 10.1038/s41467-019-11115-x (PMC6667505; doi:10.1038/s41467-019-11115-x)
Supplement: Supplementary file 1 — Supplementary Information [file 41467_2019_11115_MOESM1_ESM.pdf]

Supplementary information for:

MAPCap allows high-resolution detection and differential expression analysis of transcription start sites

Bhardwaj et al. 2019

Supplementary Figures

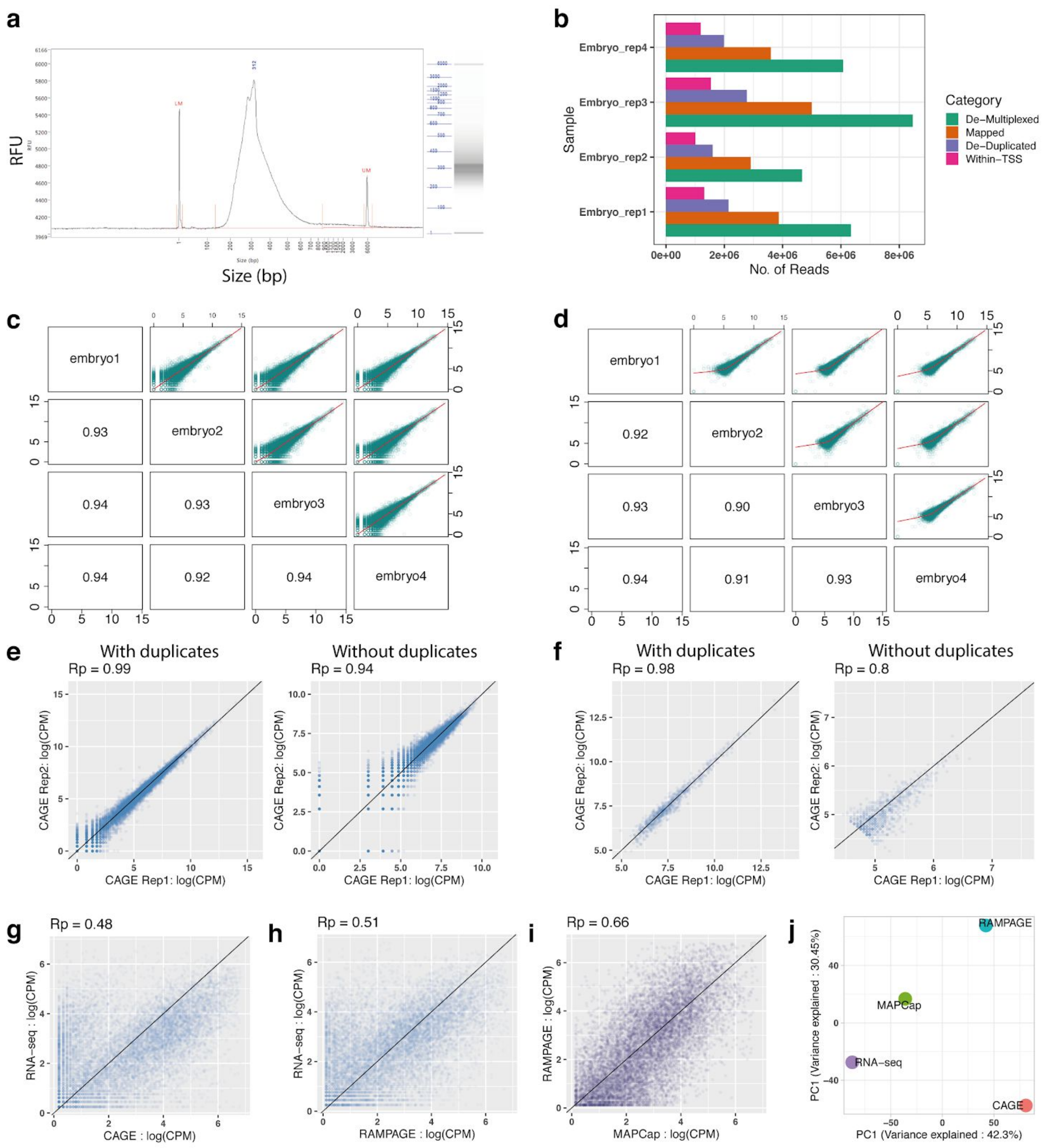

Supplementary Fig. 1. Assessment of the MAPCap data quality. a, BioAnalyzer profile of the MAPCap library (RFU = relative fluorescence unit). **b,** Number of reads kept at each step of MAPCap analysis of stage 15 embryos. **c,** Correlation of signal (log2(CPM+1)) at the 5'-UTR of genes between replicates of the MAPCap in embryos (CPM = counts per million). **d,** Correlation of signal (log2(CPM+1)) at the TSS detected using paraclu for the MAPCap in embryos. **e,** Correlation of signal (log2(CPM+1)) at the 5'-UTR of genes between 2 replicates of CAGE data (from modENCODE) in S2 cells. **f,** Correlation of signal (log2(CPM+1)) at the TSS detected using paraclu between 2 replicates of CAGE data (from modENCODE) in S2 cells. **g-i,** Same as Fig. 1c-d; correlation of signal (log2(CPM+1)) between CAGE:RNA-seq, RAMPAGE:RNA-seq and MAPCap:RAMPAGE on 5'UTRs. **j,** PCA plot showing the relationship between MAPCap, RNA-seq and other 5'-profiling protocols.

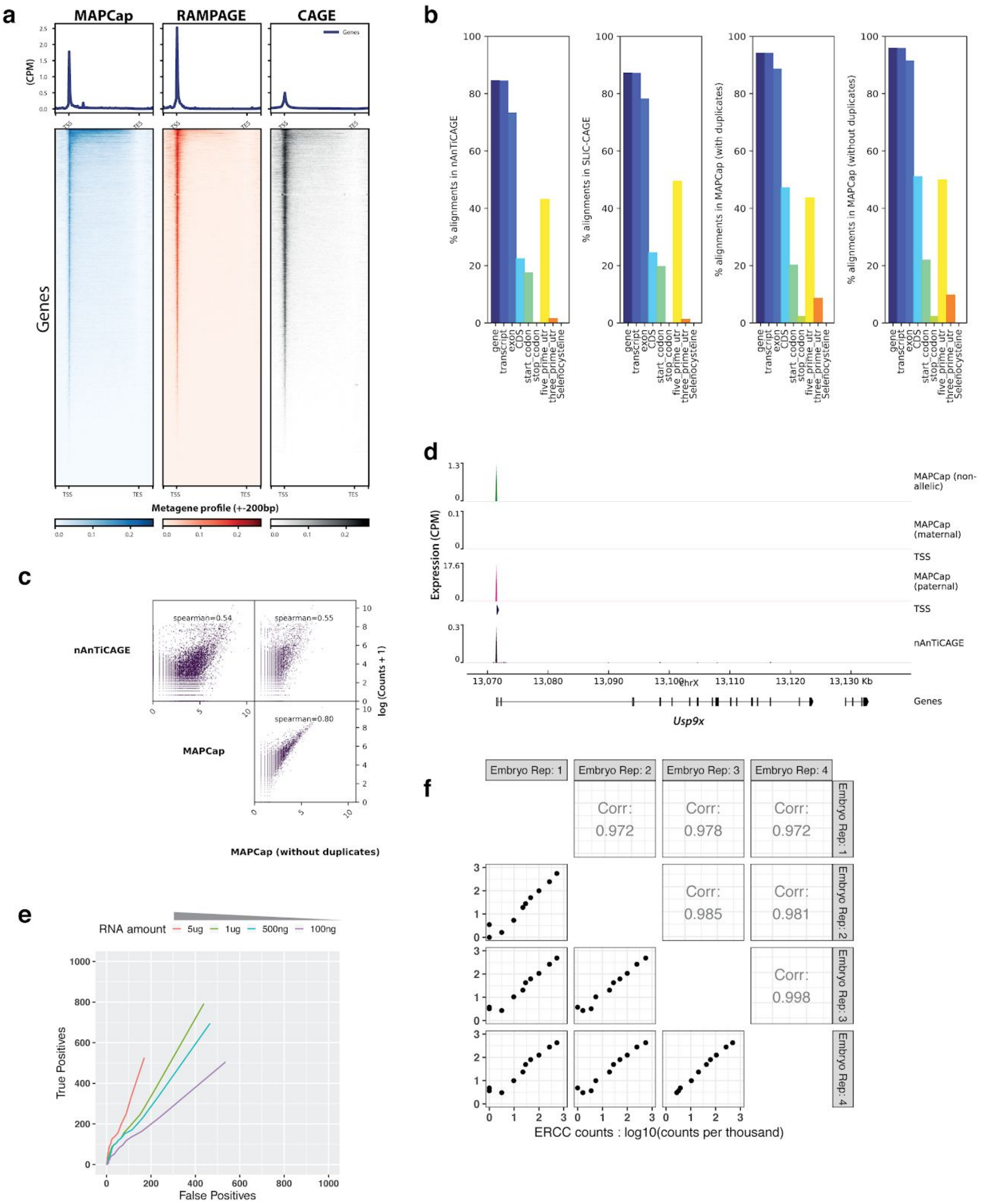
a
MAPCap
RAMPAGE
CAGE
(CPM)
Genes
TSS
TES
Metagene profile (+-200bp)
b
% alignments in nAnTiCAGE
% alignments in SLIC-CAGE
% alignments in MAPCap (with duplicates)
% alignments in MAPCap (without duplicates)
gene
transcript
exon
CDS
start_codon
stop_codon
five_prime_utr
three_prime_utr
Selenocysteine
c
nAnTiCAGE
spearman=0.54
spearman=0.55
spearman=0.80
MAPCap
MAPCap (without duplicates)
log (Counts + 1)
d
Expression (CPM)
MAPCap (non-allelic)
MAPCap (maternal)
TSS
MAPCap (paternal)
TSS
nAnTiCAGE
13,070
13,080
13,090
13,100
13,110
13,120
13,130 Kb
chrX
Genes
Usp9x
e
RNA amount
5ug
1ug
500ng
100ng
True Positives
False Positives
f
Embryo Rep: 1
Embryo Rep: 2
Embryo Rep: 3
Embryo Rep: 4
Corr: 0.972
Corr: 0.978
Corr: 0.972
Corr: 0.985
Corr: 0.981
Corr: 0.998
ERCC counts : log10(counts per thousand)

Supplementary Fig. 2. Comparison of MAPCap and other protocols. a, Metagene profiles of MAPCap, RAMPAGE, and CAGE signal (CPM) after removal of PCR duplicates, for MAPCap and RAMPAGE, PCR duplicates were removed using 5'-read position + random barcodes. **b,** Enrichment of signal at different genic regions in nAnTiCAGE, SLIC-CAGE and MAPCap in mouse ESCs **c,** Correlation of signal at known genes, between nAnTiCAGE and MAPCap (before and after duplicate removal) **d,** Allele-specific TSS detection on the paternal genome for the Usp9x gene using MAPCap data. **e,** Evaluation of TSS detected from the MAPCap experiment performed in S2 cells, using different quantity of total RNA as starting material (5ug, 1ug, 500ng, 100ng) **f,** Correlation of recovered counts of individual ERCC oligos between samples. Oligo mix was created by 2-fold serial dilution of individual oligos.

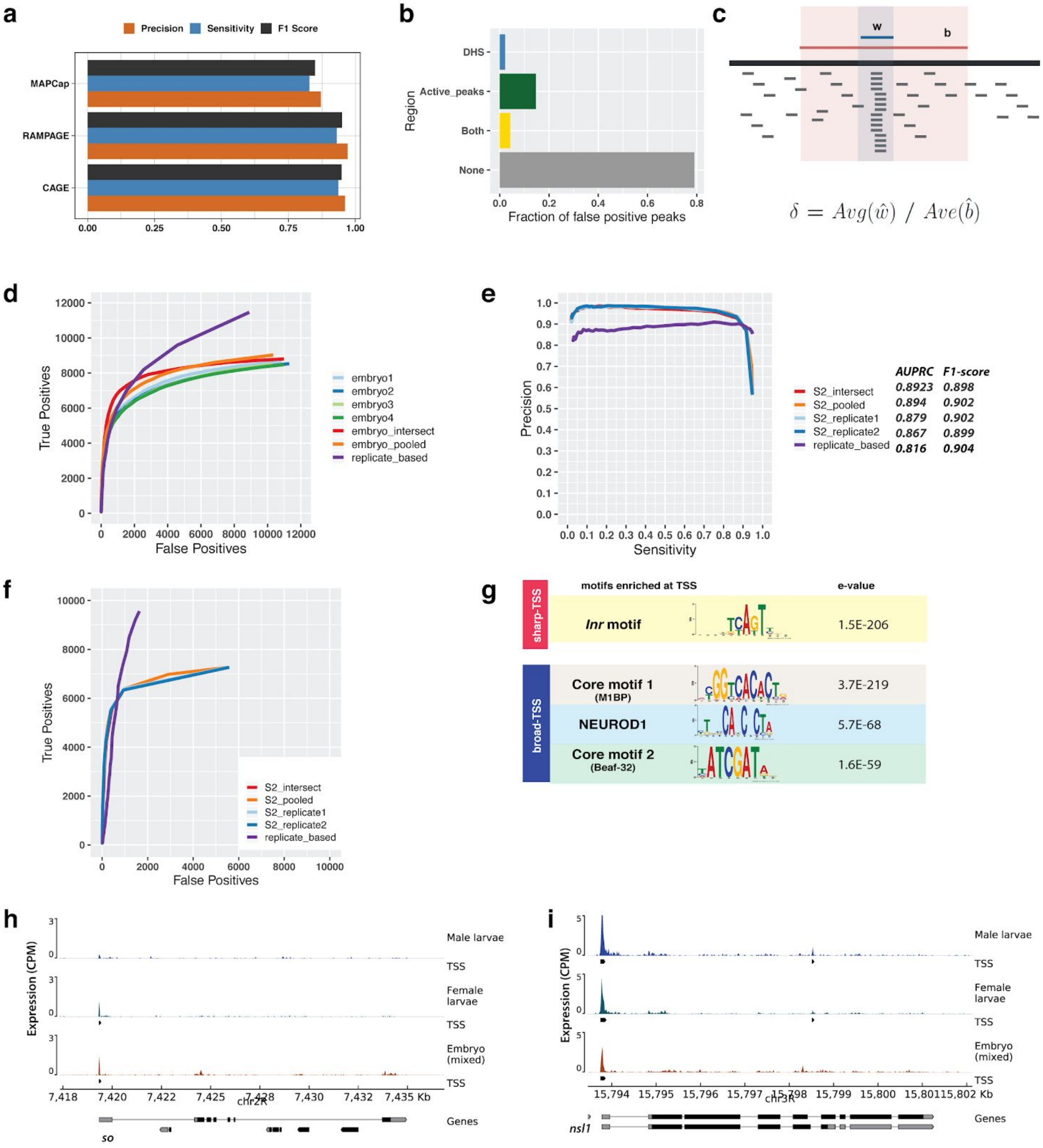
a
Precision
Sensitivity
F1 Score
MAPCap
RAMPAGE
CAGE
0.00
0.25
0.50
0.75
1.00
b
Region
DHS
Active_peaks
Both
None
0.0
0.2
0.4
0.6
0.8
Fraction of false positive peaks
c
w
b
$\delta = Avg(\hat{w}) / Ave(\hat{b})$
d
True Positives
False Positives
embryo1
embryo2
embryo3
embryo4
embryo_intersect
embryo_pooled
replicate_based
e
Precision
Sensitivity
AUPRC
F1-score
S2_intersect 0.8923 0.898
S2_pooled 0.894 0.902
S2_replicate1 0.879 0.902
S2_replicate2 0.867 0.899
replicate_based 0.816 0.904
f
True Positives
False Positives
S2_intersect
S2_pooled
S2_replicate1
S2_replicate2
replicate_based
g
motifs enriched at TSS
e-value
sharp-TSS
Inr motif
1.5E-206
broad-TSS
Core motif 1 (M1BP)
3.7E-219
NEUROD1
5.7E-68
Core motif 2 (Beaf-32)
1.6E-59
h
Expression (CPM)
Male larvae
TSS
Female larvae
TSS
Embryo (mixed)
TSS
7,418
7,420
7,422
7,425
7,428
7,430
7,432
7,435 Kb
chr2R
Genes
so
i
Expression (CPM)
Male larvae
TSS
Female larvae
TSS
Embryo (mixed)
TSS
15,794
15,795
15,796
15,797
15,798
15,799
15,800
15,801
15,802 Kb
chr3R
Genes
nsl1

Supplementary Fig. 3. Evaluation of MAPCap, along with our new TSS detection approach. a, Precision, Sensitivity and F1-score (see methods) of TSS detection using paraclu on replicate-pooled and subsampled data from MAPCap, CAGE (modENCODE) and RAMPAGE. **b,** Overlap of TSS labeled as “false positives” in the previous analysis with peaks for DNAse-seq or active histone marks. “Active peaks” are peaks enriched for H3K4me3, H3K4me1 or H3K27ac marks. **c,** Schematic diagram of “local enrichment” method, the fold-change (δ; delta) for windows (w) over background (b) is calculated as average fold change of replicates after depth-normalization. **d,** Evaluation of true and false positives from the TSS detected using the local enrichment method on MAPCap data (similar to Fig. 1a), the scores here represent the “density rise” (the difference between the highest and lowest signal on the region) instead of total no. of reads, as in Fig. 2a. **e,** Precision-recall curve of TSS detected using our method compared to paraclu, on 1 Mil subsampled reads using CAGE data (modENCODE, S2 cells). **f,** Evaluation of true and false positives from the TSS detected using the local enrichment method on CAGE data. **g,** de-novo motif enrichment results for the “sharp” and “broad” TSS. Only top motifs (e-value < 1E-50) are shown. **h-i,** Genome snapshot of MAPCap signal and the detected TSS using the “local enrichment” method on a developmental gene (so) and a housekeeping gene (Nsl1).

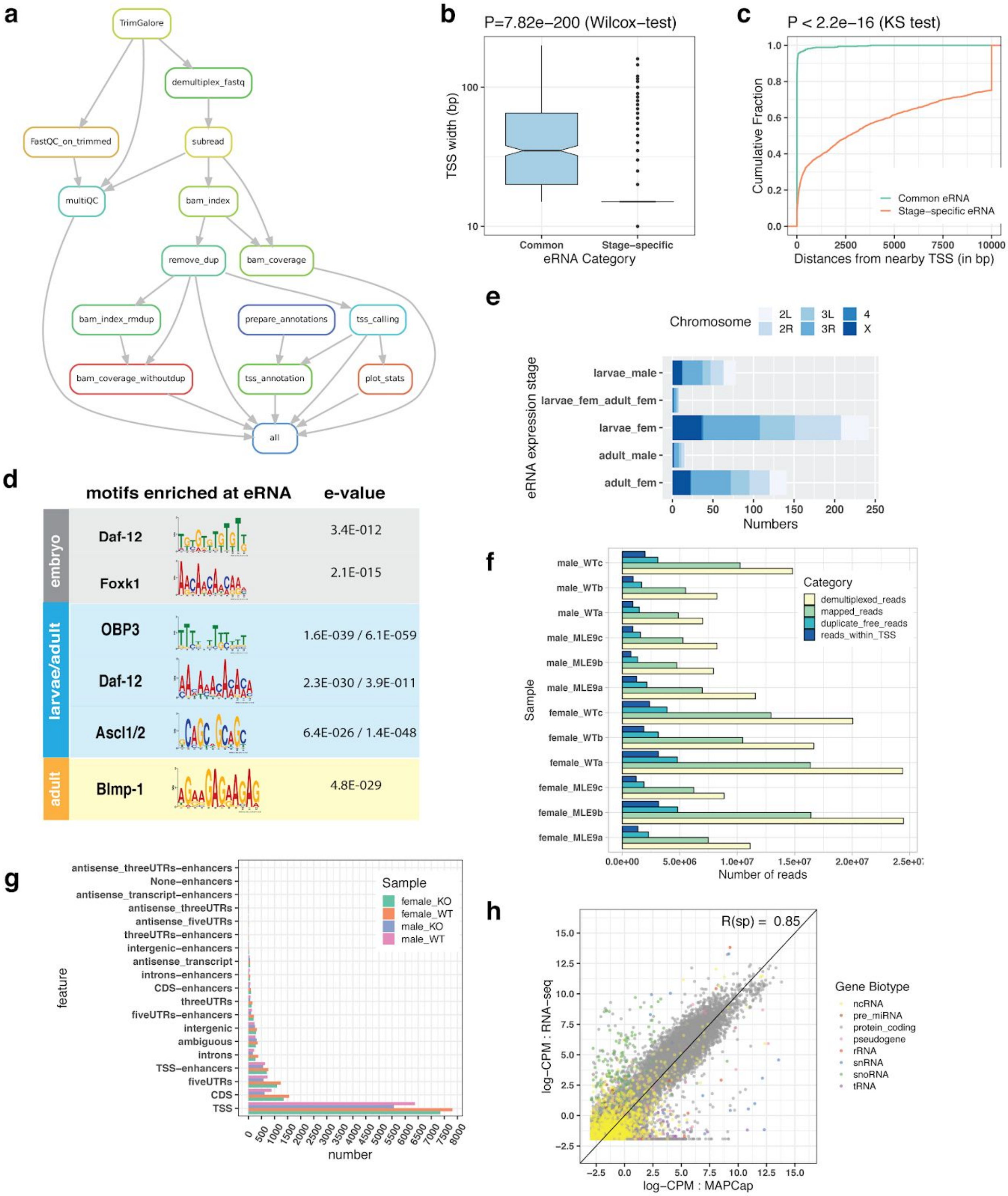
a
TrimGalore
demultiplex_fastq
FastQC_on_trimmed
subread
multiQC
bam_index
remove_dup
bam_coverage
bam_index_rmdup
prepare_annotations
tss_calling
bam_coverage_withoutdup
tss_annotation
plot_stats
all
b
P=7.82e−200 (Wilcox−test)
TSS width (bp)
100
10
Common
Stage−specific
eRNA Category
c
P < 2.2e−16 (KS test)
Cumulative Fraction
Common eRNA
Stage−specific eRNA
Distances from nearby TSS (in bp)
d
motifs enriched at eRNA
e-value
embryo
Daf-12
3.4E-012
Foxk1
2.1E-015
larvae/adult
OBP3
1.6E-039 / 6.1E-059
Daf-12
2.3E-030 / 3.9E-011
Ascl1/2
6.4E-026 / 1.4E-048
adult
Blmp-1
4.8E-029
e
Chromosome
2L
2R
3L
3R
4
X
eRNA expression stage
larvae_male
larvae_fem_adult_fem
larvae_fem
adult_male
adult_fem
Numbers
f
Category
demultiplexed_reads
mapped_reads
duplicate_free_reads
reads_within_TSS
Sample
male_WTc
male_WTb
male_WTa
male_MLE9c
male_MLE9b
male_MLE9a
female_WTc
female_WTb
female_WTa
female_MLE9c
female_MLE9b
female_MLE9a
Number of reads
g
Sample
female_KO
female_WT
male_KO
male_WT
feature
antisense_threeUTRs−enhancers
None−enhancers
antisense_transcript−enhancers
antisense_threeUTRs
antisense_fiveUTRs
threeUTRs−enhancers
intergenic−enhancers
antisense_transcript
introns−enhancers
CDS−enhancers
threeUTRs
fiveUTRs−enhancers
intergenic
ambiguous
introns
TSS−enhancers
fiveUTRs
CDS
TSS
number
h
R(sp) = 0.85
log−CPM : RNA−seq
log−CPM : MAPCap
Gene Biotype
ncRNA
pre_miRNA
protein_coding
pseudogene
rRNA
snRNA
snoRNA
tRNA

Supplementary Fig. 4. Analysis of eRNA and gene expression from MAPCap data. a, Workflow used for the analysis of data. It involves read trimming, demultiplexing using sample barcodes, mapping, duplicate removal (using random barcodes), TSS detection, TSS annotation and generation of coverage files and plots (see methods). **b,** Comparison of TSS widths of stage-specific and common eRNAs from Fig. 2g (boxplots = median and IQR). Stage-specific eRNAs have significantly sharper TSS ($p = 7.82e-200$, Wilcox test). **c,** Cumulative overlap of the fraction of detected TSSs with an annotated TSS at increasing distances. TSSs of stage-specific eRNAs are located significantly further from a known TSS. **d,** de-novo motif analysis of stage-specific eRNA TSS. **e,** Chromosomal locations of sex-specific eRNAs (from Fig. 2h). **f,** Number of reads kept at each step of the analysis. **g,** Comprehensive annotation of detected TSS in each group of samples. **h,** Comparison of the signal (log2(CPM+1)) at 5'UTRs between RNA-seq and MAPCap (similar to Fig. 3c) for wild-type female larvae brains.

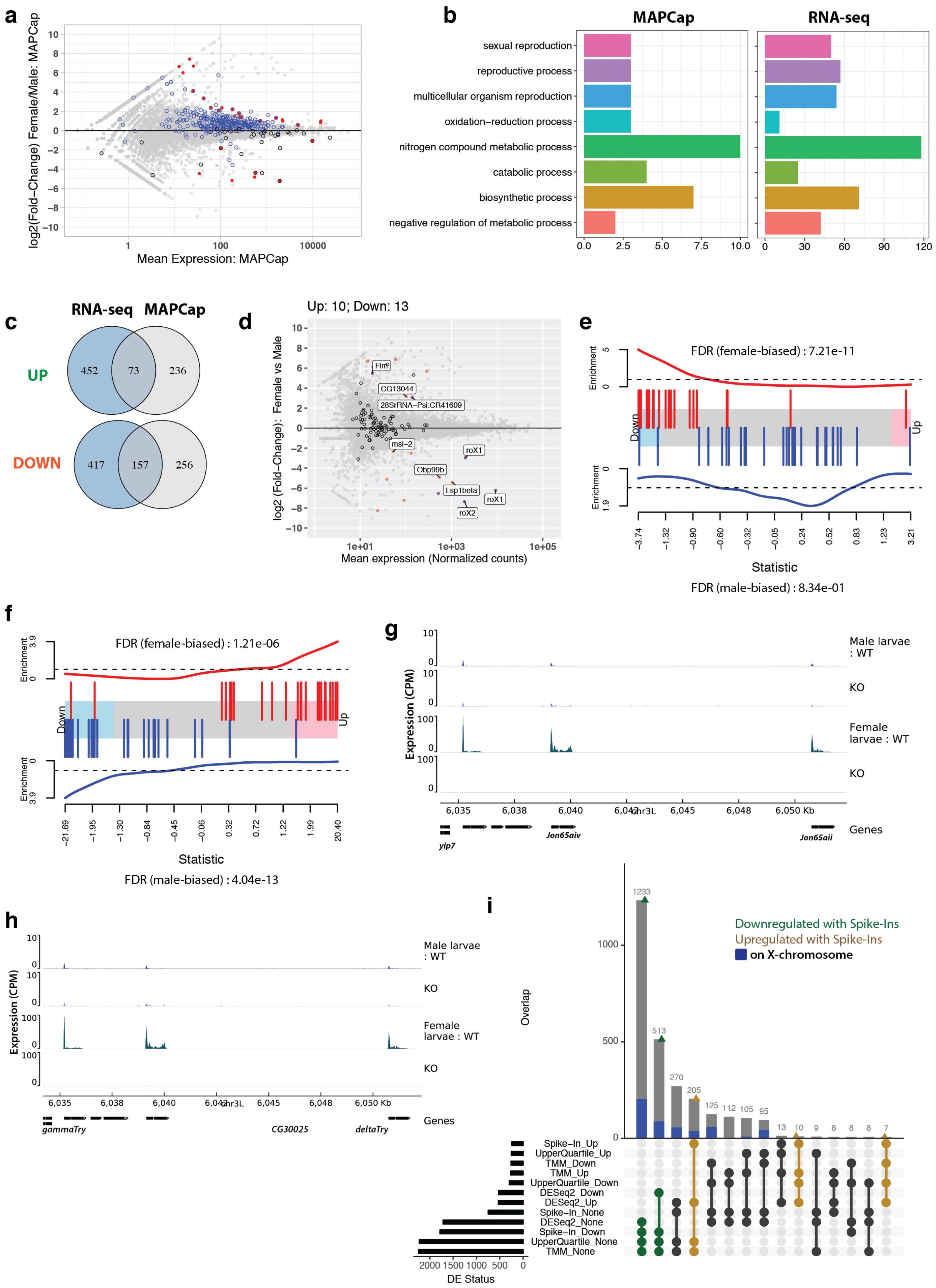

a
log2(Fold−Change) Female/Male: MAPCap
Mean Expression: MAPCap
b
MAPCap
RNA-seq
sexual reproduction
reproductive process
multicellular organism reproduction
oxidation−reduction process
nitrogen compound metabolic process
catabolic process
biosynthetic process
negative regulation of metabolic process
c
RNA-seq
MAPCap
UP
452
73
236
DOWN
417
157
256
d
Up: 10; Down: 13
log2 (Fold−Change): Female vs Male
Mean expression (Normalized counts)
Fim
CG13044
28SrRNA−Psi:CR41609
msl−2
roX1
Obp99b
Lsp1beta
roX1
roX2
e
FDR (female-biased) : 7.21e-11
Enrichment
Down
Up
Statistic
FDR (male-biased) : 8.34e-01
f
FDR (female-biased) : 1.21e-06
Enrichment
Down
Up
Statistic
FDR (male-biased) : 4.04e-13
g
Expression (CPM)
Male larvae : WT
KO
Female larvae : WT
KO
chr3L
Genes
yip7
Jon65aiv
Jon65aii
h
Expression (CPM)
Male larvae : WT
KO
Female larvae : WT
KO
chr3L
Genes
gammaTry
CG30025
deltaTry
i
Downregulated with Spike-Ins
Upregulated with Spike-Ins
on X-chromosome
Overlap
Spike−In_Up
UpperQuartile_Up
TMM_Down
TMM_Up
UpperQuartile_Down
DESeq2_Down
DESeq2_Up
Spike−In_None
DESeq2_None
Spike−In_Down
UpperQuartile_None
TMM_None
DE Status

Supplementary Fig. 5. Differential expression and sex-bias analysis in male and female larvae brains. a, 5'UTR differential expression (DE) analysis with MAPCap using DESeq2 (internal normalization). DE genes are marked in red. DE genes obtained from RNA-seq are circled in Blue (upregulated) and Black (downregulated). **b,** Go term analysis of DE genes obtained from MAPCap (left) and RNA-seq (right). **c,** Overlap of DE genes between MAPCap and RNA-seq (at FDR < 0.05) for male MLE KOs. **d,** MA-plot showing sex-biased activity of TSS between wild-type male and female brains using MAPCap. TSSs with significant sex-bias (at FDR < 0.05) are marked in red. eRNAs detected specifically in male or females are marked in black. **e-f,** barcodePlots of female and male-biased TSS over the t-statistics of differential expression in female and male MLE mutants, respectively. The female-biased genes show downregulation in MLE mutants, while male-biased genes show downregulation in male MLE mutants. **g-h,** Examples of autosomal loci that shows sexually dimorphic (female-biased) expression and are downregulated in MLE mutant (KO) flies. **i,** Overlap of differential expression results on TSSs using different normalizations (TMM, Upper Quartile, DESeq2, or Spike-in).

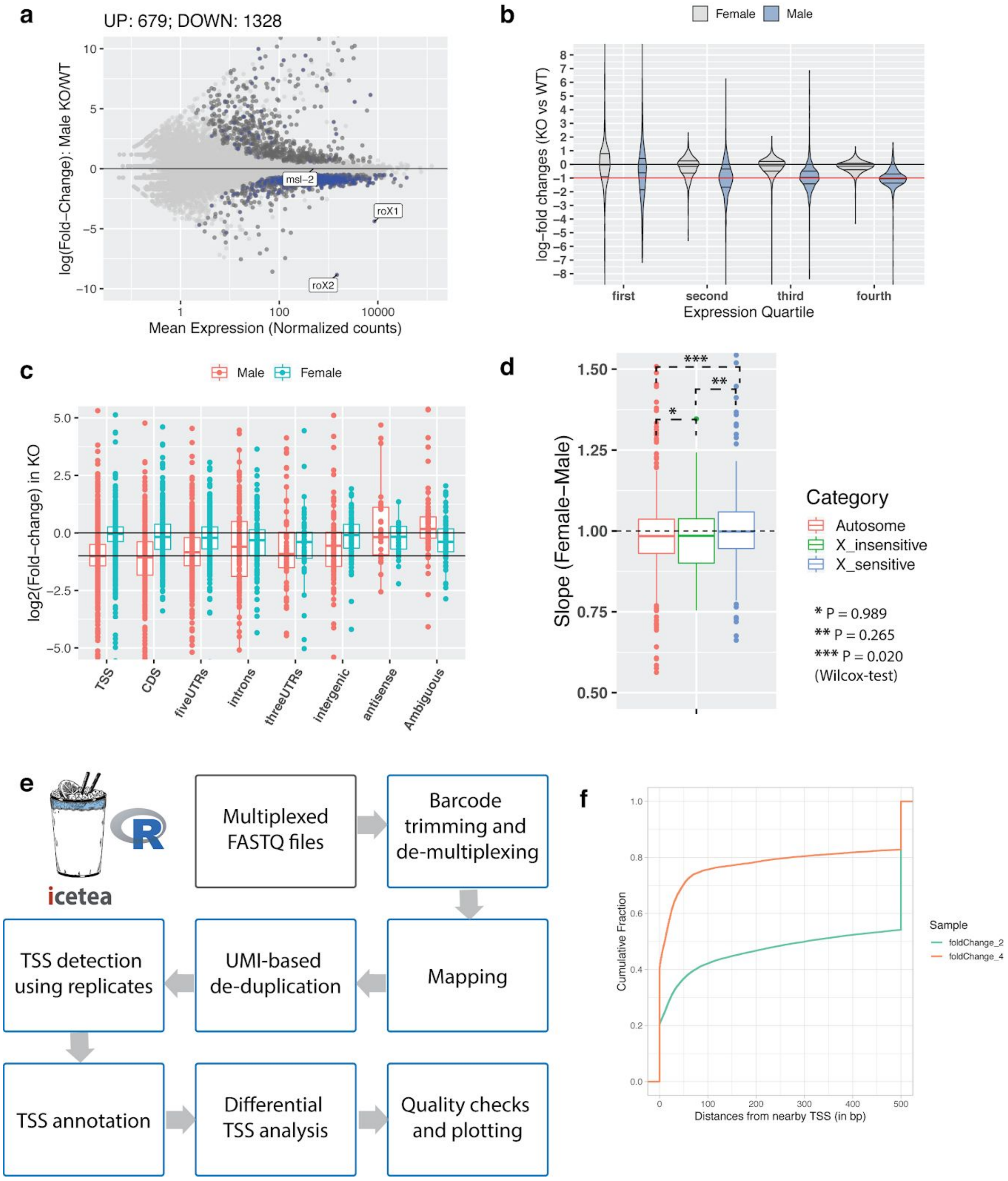

a
UP: 679; DOWN: 1328
log(Fold−Change): Male KO/WT
Mean Expression (Normalized counts)
msl−2
roX1
roX2
b
Female
Male
log−fold changes (KO vs WT)
first
second
third
fourth
Expression Quartile
c
Male
Female
log2(Fold−change) in KO
TSS
CDS
fiveUTRs
introns
threeUTRs
intergenic
antisense
Ambiguous
d
Slope (Female−Male)
Category
Autosome
X_insensitive
X_sensitive
* P = 0.989
** P = 0.265
*** P = 0.020
(Wilcox-test)
e
icetea
Multiplexed FASTQ files
Barcode trimming and de-multiplexing
Mapping
UMI-based de-duplication
TSS detection using replicates
TSS annotation
Differential TSS analysis
Quality checks and plotting
f
Cumulative Fraction
Distances from nearby TSS (in bp)
Sample
foldChange_2
foldChange_4

Supplementary Fig. 6. MLE sensitivity of promoters on the X-chromosome. a, MA plot from RNA-seq data of male MLE KOs compared to wild-type males, using DESeq2 (genes on X-chromosome are in blue). **b,** Log2-fold Changes of expression upon MLE KO, for male and female X-chromosome genes (n = 2840). Genes are divided by their wild-type expression quartiles (first = lowest, fourth = highest). **c,** Effect of MLE KO on TSSs in various locations of the X-chromosome (TSS = annotated TSS, Ambiguous = TSS overlapping multiple/neither of the locations). **d,** Comparison of the "compensation score" (female to male ratio of slope of expression) for MLE-sensitive genes identified in this study from Lott et al. (2011) [28] (n = autosome: 500, X_insensitive: 43, X_sensitive: 212, boxplots = median and IQR) **e,** Steps of data analysis implemented in icetea. Read de-multiplexing and de-duplication based on UMI is supported for MAPCap and RAMPAGE data. (R logo reused under CC-BY-SA-4.0 from https://www.r-project.org/logo/) **f,** An example of QC output from icetea: TSS distance precision (the distance of detected TSS to nearby annotated TSS) at different fold-change cutoffs on embryo data (using "plotTSSprecision" function)

Supplementary Tables

Supplementary table 1: PCR primers for spike-ins

name	sequence
T7F25f	TAATACGACTCACTATTAG
ERCC-25r	GATCTCTCTACCGCCCTAAA
ERCC-81r	GACACCCCACTGCGCGTGTG
ERCC-43r	GACTCTAAGATGATGTGGTC
ERCC-137r	GATCACCCAAAAACGAGCGC
ERCC-136r	GAGGCGTATACCCACGTCCT
ERCC-74r	GATTCTCTGCATCCTATTTA
ERCC-109r	GAACGGGCTAAAGTGAGCTT
ERCC-99r	GGACCTAAATAACCGACTTT
ERCC-145r	GACCCGTTTCTTTACATAGG
ERCC-158r	GAGTCTTCACACTACCAATA

Supplementary table 2: Antisense oligos against Dmel small abundant RNAs (sn-, snoRNAs)

Name	Sequence	Target
dmMAPCap_AS_01	CCATAAGGCCGAGAAGCGAT	snRNA:U2
dmMAPCap_AS_02	CCTCTACGCCAGGTAAGTAT	snRNA:U1
dmMAPCap_AS_03	TATCGCCTCTGCGCAAAGAT	snRNA:U4
dmMAPCap_AS_04	TATTGCCACTGCGCAAAGAT	snRNA:U4
dmMAPCap_AS_06	CAGTCTACCTCTACTAATGA	snoRNA:185
dmMAPCap_AS_07	TGAAGCGGCGATCGAGACAT	snRNA:LU
dmMAPCap_AS_08	ATCCTGTGAAGTATAGTCTT	snoRNA:Me28S
dmMAPCap_AS_09	AATTGAAGAGAAACCAGAGT	snRNA:U5
dmMAPCap_AS_10	AGAGAATAAAAATTTTCAAT	snRNA:U7
dmMAPCap_AS_11	ACCCAATCGTCACCTCTCGCA	snoRNA:Or-CD1
dmMAPCap_AS_12	CCAGGACGAGCACCCTTTTT	snRNA:U11
dmMAPCap_AS_13	CTCCCCAAGACAAGGAAGGT	snRNA:U4atac
dmMAPCap_AS_14	TACTCATTAGTTTGAGGCAC	snRNA:U12
dmMAPCap_AS_15	TTCATCATATCATCTAGAGA	snoRNA:Me18S
dmMAPCap_AS_16	TGTTCTGCCGAAGCAAGAAC	snRNA:U6
dmMAPCap_AS_17	GCTCTCCTTCCAAACAACAC	snRNA:U6atac
dmMAPCap_AS_18	ATGTAATGTTCATCATGTCG	snoRNA:Me28S

Supplementary table 3: Antisense oligos against Dmel rRNAs

Name	Sequence	Target
dmMAPCap_rRNAas_01	tactttcattgtagcgcgcg	18SrRNA:CR41548
dmMAPCap_rRNAas_02	tatatgtcatgctcttctag	28SrRNA:CR45837
dmMAPCap_rRNAas_03	gacctctcggtctaggaaat	18SrRNA:CR41548

dmMAPCap_rRNAas_04	ccattcatgcgcgtcactaa	28SrRNA-Psi:CR45851
dmMAPCap_rRNAas_05	ggctaccttaagagagtcat	28SrRNA-Psi:CR45851
dmMAPCap_rRNAas_06	ccccgctaattattccaagcc	28SrRNA-Psi:CR45851
dmMAPCap_rRNAas_07	tgtctccttacactgccaga	28SrRNA-Psi:CR45851
dmMAPCap_rRNAas_08	gggtgaacaatccaacgctt	28SrRNA-Psi:CR45851
dmMAPCap_rRNAas_09	caacgttttgtcattagtagggt	28SrRNA-Psi:CR45851
dmMAPCap_rRNAas_10	atgtaactagcgcggcatca	28SrRNA-Psi:CR45851
dmMAPCap_rRNAas_11	aaccctgattccccgttacc	18SrRNA:CR41548
dmMAPCap_rRNAas_12	tcaaagtaatagtaccggccca	18SrRNA:CR41548
dmMAPCap_rRNAas_13	tgatctgaaaaccaatgaaagca	18SrRNA:CR41548
dmMAPCap_rRNAas_14	acgacggtccaagaatttcac	18SrRNA:CR41548
dmMAPCap_rRNAas_15	actagggcggtatctgatcg	18SrRNA:CR41548
dmMAPCap_rRNAas_16	atcccaagcatgaaagtggt	18SrRNA:CR41548
dmMAPCap_rRNAas_17	tgcacatatgttagactccttgg	28SrRNA:CR45837
dmMAPCap_rRNAas_18	ccattcatgcgcgtcactaa	28SrRNA:CR45837
dmMAPCap_rRNAas_19	tgcacatatgttagactccttgg	28SrRNA-Psi:CR45851
dmMAPCap_rRNAas_20	ttttcaaggtccgaggagaaa	28SrRNA:CR45837
dmMAPCap_rRNAas_21	agtcaaactccctacctggc	28SrRNA-Psi:CR45851
dmMAPCap_rRNAas_22	cactgagctggccttggg	28SrRNA-Psi:CR45851
dmMAPCap_rRNAas_23	acaccgagatcaagtcagca	28SrRNA-Psi:CR45851
dmMAPCap_rRNAas_24	ccacaagccagttatccctatg	28SrRNA-Psi:CR45851
dmMAPCap_rRNAas_25	accaaaaggatcgataggcc	28SrRNA-Psi:CR45851
dmMAPCap_rRNAas_26	tgcggttcctctcgtactac	28SrRNA-Psi:CR45851

Supplementary table 4: External datasets used in this study

Description	Dataset Accession	Reference
**modENCODE CAGE**	**SRA**	13
embryo 12-14hr	SRR488302	
S2 cells	SRR488286-87	
adult male and female	SRR488271-72, 77-80	
**modENCODE RNA-seq**	**SRA**	93
embryo 12-14hr	SRR1197369<br>SRR1197332	
S2 cells	SRR1197280	
modENCODE ChIP-seq:<br>H3K4me3 (E12-16)<br>H3K4me1 (E12-16)<br>H3K27ac (E12-16)<br>Input (E12-16)	GEO:GSE16013	92
RAMPAGE (embryo 12-14hr)	GEO:GSE36213	3
DNAse-seq (stage-14 embryos)	SRA:SRP002474	43
DNAse-seq (S2 cells)	GEO:GSE40739	90
nAnTiCAGE (mouse ESCs)	ENA:E-MTAB-6519	7
